# Subacute Microcystin-LR Exposure Alters the Metabolism of Thyroid Hormones in Juvenile Zebrafish (*Danio Rerio*)

**DOI:** 10.3390/toxins7020337

**Published:** 2015-01-30

**Authors:** Zidong Liu, Rong Tang, Dapeng Li, Qing Hu, Ying Wang

**Affiliations:** 1 College of Fisheries, Huazhong Agricultural University, Wuhan 430070, China; E-Mails: liuzidong0202@163.com (Z.L.); huqinggw@webmail.hzau.edu.cn (Q.H.); wyingale@gmail.com (Y.W.); 2 Freshwater Aquaculture Collaborative Innovation Center of Hubei Province, Wuhan 430070, China; 3 Key Laboratory of Freshwater Animal Breeding, Ministry of Agriculture, Wuhan 430070, China; 4 Life Science College, Hunan University of Arts and Science, Changde 415000, China

**Keywords:** microcystin-LR, thyroid hormones, histology, gene expression, iodothyronine deiodinases, zebrafish

## Abstract

Microcystin-LR (MC-LR) has been detected extensively in the aquatic environment and has the potential to disturb the thyroid endocrine system. However, limited information is available on the effects of subacute MC-LR exposure on fish thyroid hormone (TH) metabolism. In the present study, juvenile zebrafish (*Danio rerio*) were exposed to MC-LR at environmentally relevant concentrations (0, 1, 5, and 25 μg/L) for 28 days. Whole-body TH content and thyroid follicle histology were used as direct endpoints to assess thyroid disruption. The activities of iodothyronine deiodinases (IDs) and the transcription of selected genes associated with TH synthesis were also investigated to study the underlying mechanisms of endocrine disruption. Exposure of zebrafish to MC-LR significantly increased whole-body thyroxine (T_4_) content but decreased whole-body triiodothyronine (T_3_) content. We also observed hypertrophy and hyperplasia of the thyroid follicle epithelial cells, as well as up-regulation of corticotropin-releasing hormone (CRH), thyroid-stimulating hormone (TSH), thyroid peroxidase (TPO), and transthyretin (TTR) genes. The decreases in ID1 and ID2 activities coupled with an increase in ID3 activity were observed in MC-LR treatment groups. These results demonstrate that exposure to MC-LR at environmental concentrations results in the disturbance of TH homeostasis by disrupting the synthesis and conversion of THs.

## 1. Introduction

Cyanobacterial blooms occur constantly worldwide and have been regarded as a serious environmental issue [1]. A potentially hazardous consequence of cyanobacterial blooms is the production of several kinds of cyanotoxins. The microcystins (MCs) are the most commonly identified toxins in freshwater blooms [2]. Despite the identification of nearly 80 structurally different MCs, microcystin-LR (MC-LR) is recognized as being the most toxic and is distributed worldwide in freshwater environments [3,4]. Although a few studies have reported that maximal dissolved MCs concentration reached to 78 μg/L [5], the environmental concentration of dissolved MCs during most cyanobacterial blooms in lakes usually range from 0.1 to 10 μg/L [6]. Of particular relevance is MC exposure in fish as they are very important components of aquatic ecosystems. Fish are easily exposed to MCs through ingestion of cyanobacterial cells or through contact with the surrounding water passively [7].

Microcystins exert profound impacts on fish, including the inhibition of growth [8], reproductive injury [9], hepatotoxic effects [10,11], kidney damage [12], as well as physiological and biochemical changes [13,14]. In recent years, studies have confirmed that MCs could disrupt endocrine systems in fish [15,16,17]. Exposure to MCs resulted in a significant up-regulation of the expression of mRNA for proopiomelanocortin and vitellogenin in zebrafish [17,18]. In addition, a few studies have shown that MCs could alter thyroid hormone (TH) levels and the expression of genes involved in the hypothalamic-pituitary-thyroid (HPT) axis in fish [15,16]. In most cases, TH homeostasis had been implicated as an important biomarker for detecting disruption of the thyroid endocrine system [19,20]. Although MC-LR can disturb the normal physiological processes of thyroid hormone metabolism in fish, the mechanisms underlying the changes in TH levels is still unclear.

The thyroid endocrine system is primarily controlled by the HPT axis, which is responsible for regulating TH dynamics by coordinating their synthesis, transport, and metabolism [21]. Synthesis of TH occurs in the thyroid follicle and thyroxine (T_4_) is the main hormone secreted. Corticotropin-releasing hormone (CRH) stimulates thyroid-stimulating hormone (TSH) secretion and regulates TH synthesis [22]. During this processes, thyroid peroxidase (TPO), a crucial enzyme for formation of T_4_ [23] and transthyretin (TTR), a specific TH transport protein in fish [24] also play key roles in TH metabolism. The conversion of T_4_ to triiodothyronine (T_3_), the biologically active form of the hormone, is catalyzed by the iodothyronine deiodinases (IDs) in the peripheral tissues [25]. The IDs play a crucial role in the metabolism and action of THs. Three types of ID have been described in fish, type I iodothyronine deiodinase (ID1), type II iodothyronine deiodinase (ID2), and type III iodothyronine deiodinase (ID3), which control the conversion of T_4_ to the more active T_3_ or to the inactive reverse triiodothyronine (rT_3_) and diiodothyronine (T_2_) [26].

Previous studies have demonstrated that environmental contaminants can affect the thyroid endocrine system at different sites, including TH synthesis, TH transport, and modification of ID activity [27,28,29]. The activity of IDs is a sensitive biomarker for thyroid disruption in fish that have been exposed to environmental contaminants [30,31]. To date, no studies have investigated the effects of MCs on ID activity, despite the observed changes in ID gene expression in fish exposed to sub-lethal or lethal doses of MC-LR [16]. However, changes at genes transcription level may not really reflect the variations at a functional level. Therefore, it is necessary to study the functional molecules* i.e.*, the enzymes in the cells of the organisms to make realistic conclusions [32]. In addition, the concentrations of MCs applied in previous studies of the effects of MCs on thyroid hormone disruption are rarely observed in nature. Thus, juvenile zebrafish were treated with environmentally relevant concentrations of MC-LR in order to determine whether this exposure had the potential for disruption of the thyroid system. The concentrations of T_4_ and T_3_, as well as the levels of physiologically relevant free thyroxine (FT_4_) and free triiodothyronine (FT_3_), were measured by enzyme-linked immunosorbent assay (ELISA). The changes of nuclear size in thyroid follicle epithelial cells, the activities of IDs and the expression of several genes involved in TH biosynthesis and transport were measured. The analysis of this combination of factors involved in TH metabolism allows for a more complete assessment of the mechanisms of thyroid disruption in juvenile zebrafish exposed to MC-LR.

## 2. Results

### 2.1. Whole-Body Thyroid Hormone Levels

**Figure 1:**
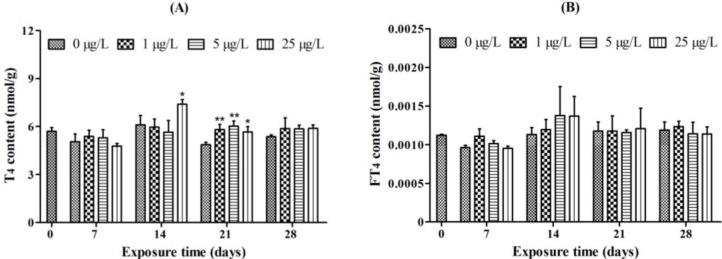

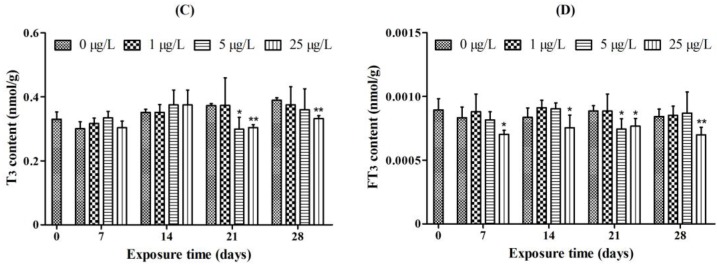
Whole-body content of (**A**) thyroxine (T_4_); (**B**) free thyroxine (FT_4_); (**C**) triiodothyronine (T_3_); and (**D**) free triiodothyronine (FT_3_) in juvenile zebrafish after exposure to different concentrations of microcystin-LR (MC-LR) (0, 1, 5, and 25 μg/L) for 7, 14, 21, and 28 days. The values are expressed as mean ± SD (*n* = 3). Significant differences obtained by one-way ANOVA followed by least significant difference (LSD) test are indicated between control and exposed groups, * *p* < 0.05, ** *p* < 0.01.

Whole-body T_4_ concentration was significantly increased in the 25 μg/L MC-LR exposure group as compared to the control group after 14 days of exposure. After 21 days of exposure, the T_4_ concentration was significantly increased in all of the MC-LR exposure groups. The T_4_ concentration gradually returned to control level in all of the exposure groups after 28 days of exposure (Figure 1A). However, there were no significant differences in the FT_4_ levels among MC-LR treatment groups (Figure 1B).

Whole-body T_3_ concentration was significantly decreased in the groups exposed to 5 or 25 μg/L MC-LR for 21 days, and to 25 μg/L MC-LR for 28 days (Figure 1C). The concentration of FT_3_ was significantly decreased after exposure to 5 and 25 μg/L MC-LR for 21 days, or to 25 μg/L MC-LR for 7, 14, and 28 days (Figure 1D).

Subacute exposure of MC-LR significantly decreased the whole-body contents of FT_3_ and T_3_. The significant fluctuations in FT_4_, however, did not occur in all groups, whereas the significant transient increase in T_4_ level was observed in the MC-LR treatment groups.

### 2.2. Histopathology

**Figure 2:**
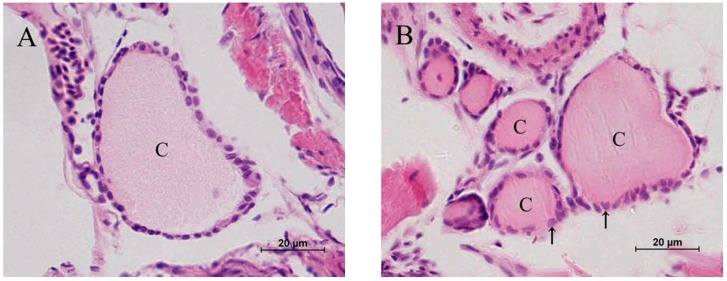

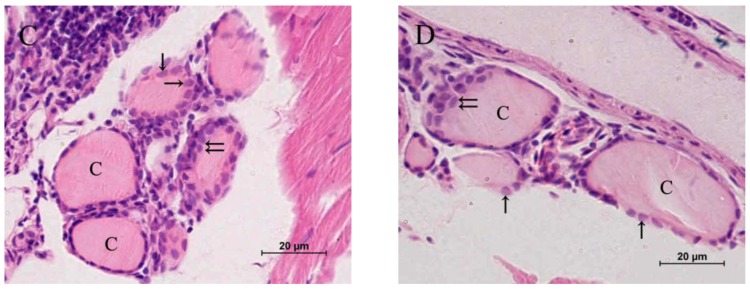
The effects of exposure to different concentrations of microcystin-LR (MC-LR) for 28 days on the histological structure of thyroid follicles in juvenile zebrafish; (**A**) Control; (**B**) 1 μg/L MC-LR; (**C**) 5 μg/L MC-LR; and (**D**) 25 μg/L MC-LR, (C in figures = colloid; single arrows = hypertrophy; double arrows = hyperplasia).

**Figure 3:**
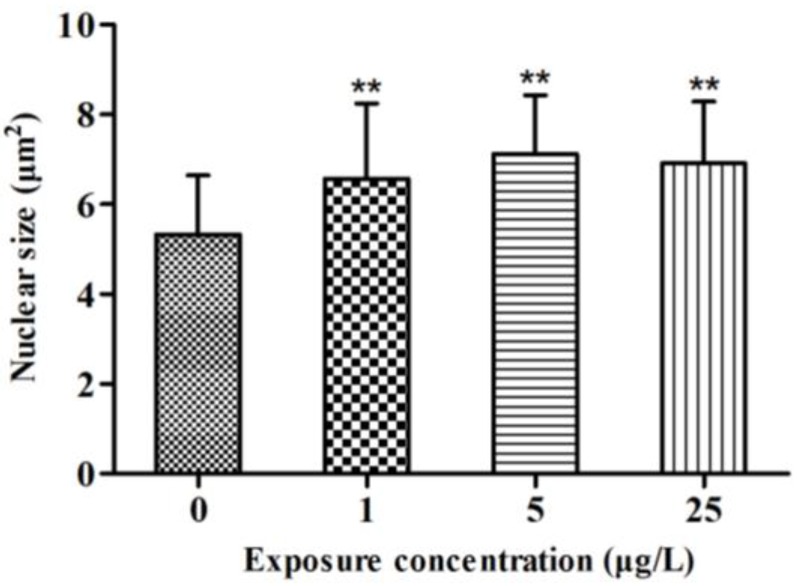
Thyroid follicle cell nuclear size following exposure of juvenile zebrafish to microcystin-LR (MC-LR) for 28 days (*n* = 3). Long and short nuclear diameters were used to estimate the cross-sectional area of at least 35 follicle cell nuclei per fish. Significant differences obtained by one-way ANOVA followed by least significant difference (LSD) test are indicated between control and exposed groups, ** *p* < 0.01.

**Figure 4:**
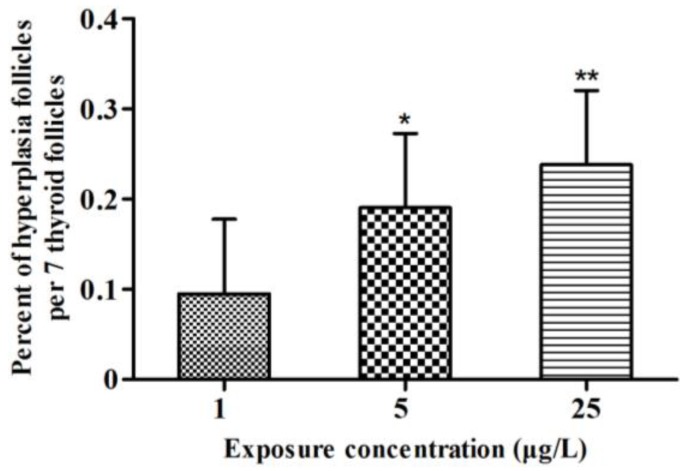
Percent of hyperplasia follicles in juvenile zebrafish exposed to microcystin-LR (MC-LR) for 28 days (*n* = 3). Significant differences obtained by one-way ANOVA followed by least significant difference (LSD) test are indicated between control and exposed groups, * *p* < 0.05, ** *p* < 0.01.

The control fish presented oval thyroid follicles consisting of an outer thyroid epithelial layer surrounding an inner lumen filled with colloid (Figure 2A). In fish treated with 1 μg/L MC-LR, the thyroid follicles had clear morphological alterations (Figure 2B). The analysis of nuclear size revealed that MC-LR significantly induced nuclear hypertrophy (Figure 2B, Figure 2C, and Figure 2D: single arrows and Figure 3). Similar results were also found in fish treated with 5 and 25 μg/L MC-LR, and hyperplasia of the thyroid follicle epithelial cells were observed (Figure 2C and Figure 2D: double arrows). Moreover, the percent of hyperplasia follicles in juvenile zebrafish indicated the significant hyperplasia in the 5 and 25 μg/L MC-LR treatment groups after 28 days of exposure (Figure 4).

The morphological results showed that hypertrophy and hyperplasia of the thyroid follicle epithelial cells of juvenile zebrafish suffered from the subacute exposure of MC-LR.

### 2.3. Gene Transcription Profile

**Figure 5:**
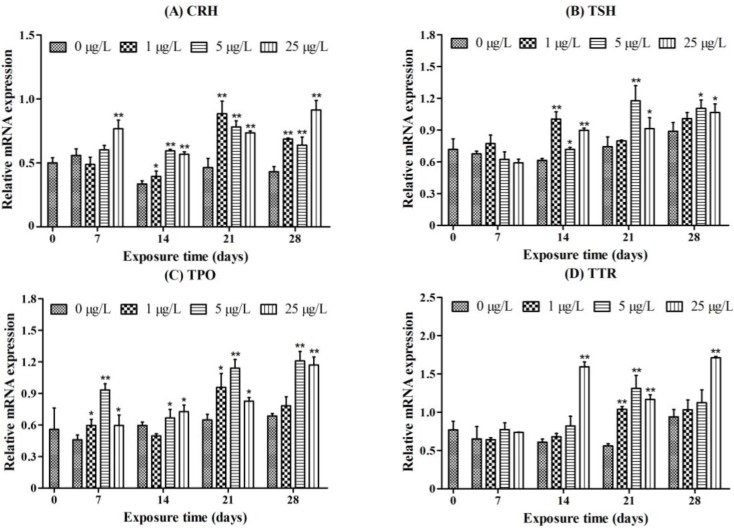
Transcript abundance for (**A**) corticotropin-releasing hormone (CRH); (**B**) thyroid-stimulating hormone (TSH); (**C**) thyroid peroxidase (TPO); and (**D**) transthyretin (TTR) in juvenile zebrafish after exposure to different concentrations of microcystin-LR (MC-LR) (0, 1, 5, and 25 μg/L) for 7, 14, 21, and 28 days. The values are expressed as mean ± SD (*n* = 3). Significant differences obtained by one-way ANOVA followed by least significant difference (LSD) test are indicated between control and exposed groups, * *p* < 0.05, ** *p* < 0.01.

The transcription of the CRH gene in the 25 μg/L MC-LR treatment group was significantly increased after seven days of exposure. Moreover, after 14, 21, and 28 days of exposure, a significant increase in the transcription of CRH was detected in all of the MC-LR treatment groups (Figure 5A).

The transcription of TSH was significantly increased at 14 day in the 1 μg/L MC-LR treatment group. In the 5 and 25 μg/L MC-LR treatment groups, the transcription of TSH was significantly increased at 14, 21, and 28 days (Figure 5B).

A significant increase in TPO transcript abundance was observed in all of the MC-LR treatment groups after 7 and 21 days of exposure. After 14 and 28 days exposure, distinct increases were seen in the 5 and 25 μg/L MC-LR treatment groups (Figure 5C).

Treatment with the high dose of MC-LR (25 μg/L) significantly increased TTR mRNA levels compared with the control group after 14, 21, and 28 days of exposure. Moreover, in the 1 and 5 μg/L MC-LR treatment groups, the transcription of TTR was also significantly increased at 21 days (Figure 5D).

In sum, subacute exposure to MC-LR, especially in the 5 or 25 μg/L MC-LR treatment groups, caused a significant up-regulation of the genes involving TH synthesis.

### 2.4. Iodothyronine Deiodinase Activities

**Figure 6:**
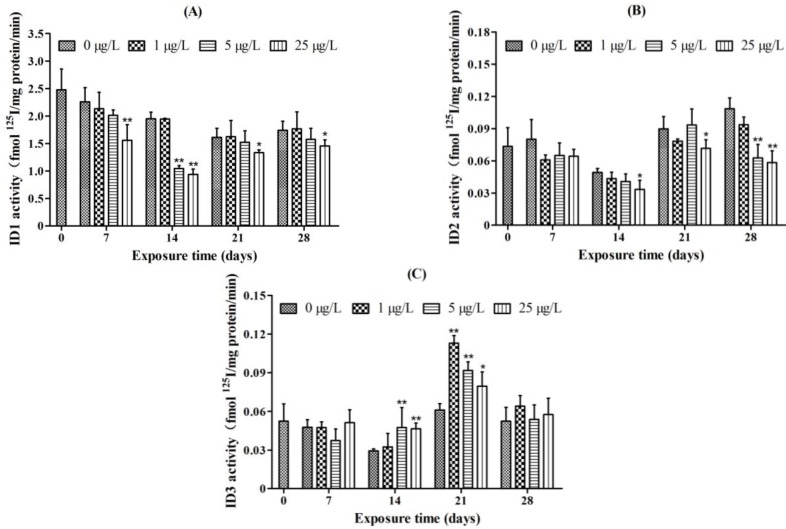
The activity of (**A**) type I iodothyronine deiodinase (ID1); (**B**) type II iodothyronine deiodinase (ID2); and (**C**) type III iodothyronine deiodinase (ID3) in juvenile zebrafish after exposure to different concentrations of microcystin-LR (MC-LR) (0, 1, 5, and 25 μg/L) for 7, 14, 21, and 28 days. The values are expressed as mean ± SD (*n* = 3). Significant differences obtained by one-way ANOVA followed by least significant difference (LSD) test are indicated between control and exposed groups, * *p* < 0.05, ** *p* < 0.01.

The activity of ID1 was significantly decreased in the 25 μg/L MC-LR treatment group after exposure for 7, 21, and 28 days. After exposure for 14 days, ID1 activity was significantly decreased in both the 5 and 25 μg/L MC-LR treatment groups (Figure 6A).

The activity of ID2 was significantly decreased in the 25 μg/L MC-LR treatment group after 14 and 21 days of exposure. After 28 days of exposure, ID2 activity was significantly decreased in both the 5 and 25 μg/L MC-LR treatment groups (Figure 6B).

The significant increase in ID3 activity was observed in the 5 and 25 μg/L MC-LR treatment groups after 14 days of exposure. After 21 days of exposure, ID3 activity was significantly increased in all of the MC-LR treatment groups (Figure 6C).

In short, subacute exposure of MC-LR reduced the activities of both ID1 and ID2. ID3 activity, however, was increased in the zebrafish exposure to MC-LR.

## 3. Discussion

In this study, treatment with environmentally relevant concentrations of MC-LR significantly altered whole-body T_4_ and T_3_ levels, the enzymatic activities of IDs, and the transcription of selected genes associated with TH synthesis. In addition, the histological changes were also observed in thyroid follicle epithelial cells. Thus, our results suggest that juvenile zebrafish exposed to environmentally relevant concentrations of MC-LR suffer physiological stress. The stress response may be implicated in the observed disruption of thyroid hormones metabolism.

The changes in both TH levels and thyroid follicle histology have typically been used as direct endpoints to assess thyroid disruption in previous studies [33]. In this study, a significant increase in T_4_ level and decrease in T_3_ level were observed after the exposure of MC-LR. Treatment with MC-LR for 14, and 21 days significantly increased whole-body T_4_ concentration, however, no significant change in the level of T_4_ was observed after exposure for 7, and 28 days. Compare with a minor transient increase in T_4_ level, T_3_, and FT_3_ significantly decreased at each sample day. It seems that MC-LR exerts deleterious effect on T_3_ production. In fish, T_4_ was the only thyroid hormone secreted in the thyroid follicle [34]. A decrease in T_3_ levels is mostly due to a decline in T_4_ production or changes in peripheral TH metabolism [15]. Our results suggest that the decrease in T_3_ levels is possibly due to the changes in peripheral TH deiodination or metabolism. IDs are crucial regulators of the concentrations of peripheral circulating THs in fish. Each ID can catalyze the removal of iodine atoms from either the outer or inner ring of THs, converting these hormones to more or less active forms. Both ID1 and ID2 are capable of converting T_4_ into T_3_, whereas ID3 is an inactivating enzyme which converts T_4_ and T_3_ to rT_3_ and T_2_, respectively [26]. Both ID1 and ID2 activities were significantly decreased in the 5 and 25 μg/L MC-LR treatment groups. Since ID1 catalyzes the conversion of T_4_ into T_3_, its decrease would contribute to a reduction in T_3_ level. ID2 exclusively catalyzes outer-ring deiodination of TH and plays a pivotal role in the production of T_3_ [23]. Therefore, decrease in ID2 activity primarily contributes to the decline of T_3_ levels. Except the disruption of the enzymatic TH activation pathway and the production of T_3_, the inactivation pathways of TH degradation were also affected by MC-LR. ID3 activity was significantly increased after MC-LR administration for 14 or 21 days. ID3 plays a vital role in TH degradation, catalyzing the conversion of T_4_ to rT_3_ and of T_3_ to T_2_. A rise in ID3 activity can result in a decrease in T_3_ level. The observed changes in the activities of all three IDs could contribute to reduced T_3_ level in MC-LR treatments, especially in the 5 and 25 μg/L MC-LR treatment groups. MC-LR can alter the IDs activities, which in turn cause a decline in T_3_ production.

Previous studies have reported that acute exposure of MC-LR led to the decrease of T_3_ and T_4_ concentrations in zebrafish embryo [16]. However, the T_4_ concentration was significantly increased at 14 day and 21 day of MC-LR exposure. And at the end of the exposure, compared to the control group, the concentration of T_4_ did not change in the treatment group. This result may be attributed to compensation regulated by the HPT axis. Unlike the mammalian thyroid endocrine system, the fish thyroid endocrine system is not centrally driven by the HPT axis [35]. In fish, CRH is regarded as a common regulator of the thyroid and adrenal/interrenal axes, controlling both ACTH and TSH release [22]. In the present study, both CRH and TSH gene transcription were significantly up-regulated after MC-LR exposure. An elevation in CRH production usually triggers an increase in TSH secretion, resulting in hypertrophy and hyperplasia of the thyroid tissue [36,37]. Changes in the level of TSH mRNA related to an altered T_4_ level have been reported in fish [38]. The synthesis and release of TH from the thyroid follicle is stimulated by TSH [39], and both T_4_ and T_3_ have negative feedback effects on TSH secretion by the pituitary in fish [40]. Therefore, the increased mRNA expressions of CRH and TSH are attributed to the negative feedback from the hypothalamus and pituitary due to the decreased levels of T_3_ [41]. In turn, the increase in CRH and TSH mRNA expressions might lead to an elevated synthesis of T_4_ in thyroid follicle. Moreover, a hyper-stimulation of thyroid follicle was observed in juvenile zebrafish after MC-LR exposure, indicating that the excessive TSH stimulates thyroid and results in hypertrophy and hyperplasia of the thyroid follicle epithelial cells. The hypertrophy of the thyroid follicle epithelial cells was also observed in 1 μg/L MC-LR treatment group. Thus, except for the negative feedback regulation, the stress response was induced by MC-LR may contribute to the increase of CRH and TSH level, leading to a hyper-stimulation of thyroid follicle [15,42]. Comparing the concentration-course patterns of hypertrophy and hyperplasia, the hypertrophy was observed in all of the MC-LR treatment groups after 28 days of exposure. However, the hyperplasia only occurred at higher concentrations of MC-LR. Liu* et al.* suggests that hypertrophy is a sensitive histopathological indicators to thyroid hormone disruption [43]. Given the significant hypertrophy observed in this study, it is inferred that thyroid hormone disruption occurred in juvenile zebrafish exposed to environmentally relevant concentrations of MC-LR. In addition, our results showed the non-linear dose-response relationship in the changes of T_4_ as well as other hormones. Compared with the acute exposure, the concentrations in this study are relatively low. Especially in the 1 or 5 μg/L MC-LR exposure group, MC-LR did not cause the serious damage to fish. Thus, fish may have ability to adapt the stress caused by MC-LR through their feedback regulation system [44,45], and frequently show non-linear dose-response relationship.

The regulatory mechanisms involving in TH synthesis are complex and act at several steps in the thyroid system. TPO is a crucial enzyme for the formation of T_4_. Increased activity of TPO has been associated with an increase in T_4_ production [46]. Thus, it seems that the up-regulation of TPO gene transcription after exposure to MC-LR could be a possible mechanism for the increased levels of T_4_.

Transthyretin (TTR) has been reported as a TH-binding and transport protein in fish [24], which can non-covalently bind most THs in the blood and regulate free TH levels [47,48]. It plays a key role in maintaining the peripheral storage of TH and regulates the supply of TH to different target tissues [49,50]. The differences in the results of TH and free TH levels may be attributable to the changes in the mRNA expression of TTR induced by MC-LR. The decreased T_3_ levels and up-regulation in TTR mRNA expression in this study appeared to lead to a more serious decreased in FT_3_ levels in this study. The differences in the results of T_4_ and FT_4_ levels may be also attributable to the elevated expression of TTR induced by MC-LR. Free TH may represent the diffusible and physiologically relevant TH fractions in the blood because the bound TH could not enter cells to elicit a response [44]. Thus, the significant decrease in FT_3_ level indicates that the juvenile zebrafish are in a hypothyroidism state after exposure to environmentally relevant concentrations of MC-LR.

## 4. Materials and Methods

### 4.1. Chemicals and Fish

Microcystin-LR (MC-LR, purity ≥95%) was purchased from Enzo Life Sciences (Lausen, Switzerland). Healthy 1-month-old juvenile zebrafish (*Danio rerio*) used in this study originated from the Institute of Hydrobiology, Chinese Academy of Sciences, Wuhan, China. All other chemicals used in this study were analytical grade.

### 4.2. Experimental Design

The stock solution of MC-LR was prepared by dissolving the toxin in dimethyl sulfoxide (DMSO, Sigma-Aldrich, St. Louis, MO, USA). A range of different concentrations for exposure (0, 1, 5, and 25 μg/L) were prepared by diluting the stock solution into dechlorinated tap water. The final concentration of DMSO in aquarium water for control and treatment groups was 0.001% (*v*/*v*).

Fish were maintained in glass tanks with dechlorinated tap water at a constant temperature (25 °C ± 1 °C) under a 14 h light/10 h dark photoperiod for 15 days in our laboratory before the start of the exposure. During the experimental exposure, fish were distributed randomly across forty-eight glass tanks (20 fish/tank) and assigned to the following treatments: three tanks for each of the exposure concentrations (0, 1, 5, and 25 μg/L MC-LR) exposed for 7, 14, 21, and 28 days, respectively. One third of the exposure solution in each tank was renewed with fresh solution containing the appropriate concentration of MC-LR every day. A commercial ELISA kit for microcystin-LR detection purchased from J & Q Environmental Technologies Co., Ltd. was used to monitor the MC-LR concentration in the tanks. The MC-LR concentration in each tank was measured, respectively, after 0, 3, 6, 9, 14, 21, and 28 days exposure. The results indicated that there were no significant differences between the target doses and measured MC-LR concentrations in the tanks during the experimental period (Table 1). The control group received 0.001% (*v*/*v*) DMSO with no MC-LR. Less than 10% mortality was observed in all of the treatments during experimentation.

**Table 1:** Measure concentrations of microcystin-LR (MC-LR) in the solutions.

Items	MC-LR concentrations (μg/L)			
Target doses of MC-LR	control	1.0	5.0	25.0
Measured MC-LR in solutions	0.00	0.88 ± 0.06	4.30 ± 0.35	23.29 ± 0.79

Data are denoted as mean ± SD.

Fish from the control and treatment tanks were sampled on experimental days 0, 7, 14, 21, and 28. The sampled fish in each experimental group were anesthetized with tricaine methanesulfonate (MS222, Sigma-Aldrich, St. Louis, MO, USA), and the heads were severed from nine fish (3 fish/tank) and fixed in Bouin’s fixative for histological examination. The rest of whole fish were immediately frozen in liquid nitrogen and stored at −80 °C for further analysis.

### 4.3. Thyroid Hormone Extraction and Measurement

Thyroid hormone measurement was performed as described by Yu* et al.* [23]. The whole-body concentrations of T_4_, FT_4_, T_3_, and FT_3_ were measured using commercial ELISA kits purchased from Beijing North Institute of Biotechnology, Beijing, China. The ELISAs for T_4_, FT_4_, T_3_, and FT_3_ were validated for use with zebrafish samples by demonstrating parallelism between a series of diluted and spiked samples in relation to the standard curve. The assay sensitivities were 12.8 nmol/L, 1.08 pmol/L, 0.38 nmol/L, and 0.38 pmol/L, for T_4_, FT_4_, T_3_, and FT_3_, respectively.

### 4.4. Histology

After fixation in Bouin’s fixative for 48 hours, the samples were washed in water and stored in 70% ethanol. The zebrafish heads were embedded in paraffin and serial transverse cross-sections (5 μm) were made using a microtome (Leica RM 2135, Heidelberg, Germany). Dewaxed and rehydrated sections were stained with hematoxylin and eosin. The size of the nucleus of the thyroid follicle cells was assessed quantitatively by measuring the long and short diameters of the cell nuclei. In one tank, 3 fish were used to analysis the histology of thyroid follicles. Thus, total 9 fish were randomly selected from each exposure group in three replicate tanks. The size of at least 35 thyroid follicle cell nuclei, from a total of 7 follicles (5 cells per follicle) from each fish, was determined using a Nikon 80i Microscope (Nikon, Tokyo, Japan). The nuclear size was calculated based on the formula for an ellipse (long diameter × short diameter × π/4) [37]. In addition, the number of thyroid follicles with characteristics of hyperplasia were counted to calculate the percent of hyperplasia follicles [43]. Photographs were taken with NIS-Element BR 3.0 software (Nikon Instruments Inc., Melville, NY, USA). 

### 4.5. Gene Expression

#### 4.5.1. RNA Extraction and Reverse Transcription

Total RNA was extracted from the whole body of juvenile zebrafish using RNAiso Plus (TaKaRa, Dalian, China). The RNA quality in each sample was assessed from the A_260/280_ ratio, as well as by 1% agarose formaldehyde gel electrophoresis. The concentration of RNA was determined using a Nanodrop 2000C Spectrophotometer (Thermo Scientific, Waltham, MA, USA). The removal of genomic DNA and the reverse transcription reaction were performed using the PrimeScript^®^ RT reagent Kit with gDNA Eraser (TaKaRa, Dalian, China) following the manufacturer’s instructions.

**Table 2:** Primers used for the quantification of the mRNA expression by real-time PCR.

Gene	Sequence of the primers (5ʹ→3ʹ)	Genbank accession No.
GAPDH	F: CTGGTGACCCGTGCTGCTT	NM001115114
	R: TTTGCCGCCTTCTGCCTTA	
CRH	F: TTCGGGAAGTAACCACAAGC	NM001007379
	R: CTGCACTCTATTCGCCTTCC	
TSH	F: GCAGATCCTCACTTCACCTACC	AY135147
	R: GCACAGGTTTGGAGCATCTCA	
TPO	F: GCGCTTGGAACACAGTATCA	EU267076
	R: CTTCAGCACCAAACCCAAAT	
TTR	F: CGGGTGGAGTTTGACACTTT	BC081488
	R: GCTCAGAAGGAGAGCCAGTG	

Abbreviations: GAPDH, glyceraldehyde-phosphate dehydrogenase; CRH, corticotropin-releasing hormone; TSH, thyroid-stimulating hormone; TPO, thyroid peroxidase; TTR, transthyretin.

#### 4.5.2. Real-Time PCR

Real-time PCR was performed in a Rotor-Gene 6000 Rotary Analyzer (Qiagen, Hilden, Germany) using SYBR^®^ Premix Ex Taq™ II (TaKaRa, Dalian, China). The forward and reverse primer sequences are listed in Table 2. The mRNA expression level of each target gene was normalized to glyceraldehyde-phosphate dehydrogenase (GAPDH) mRNA expression. The GAPDH gene was chosen as the internal control as the mRNA expression of GAPDH did not vary between control and treatment exposure groups. The thermal cycling conditions were as follows: An initial denaturation step at 95 °C for 30 s, followed by 35 cycles of 95 °C for 5 s, 55 °C for 30 s, and 72 °C for 30 s. All of the samples were analyzed in triplicate. Dissociation curve analysis was performed for each gene to check the specificity of PCR products. The mRNA expression level of each gene was calculated by the 2^−ΔΔCt^ method [51].

### 4.6. Deiodinase Activity Assays

Whole juvenile zebrafish were homogenized in buffer solution (0.01 M PBS, 1 mM DTT, 2 mM EDTA, pH 7.0) and centrifuged at 12,000× *g* for 20 min at 4 °C. The protein content of the supernatant was determined using a Bradford Protein Assay (Bio-Rad, Hercules, CA, USA). The ID activities in the supernatants were measured as previously described [52,53]. The activity of ID1 was measured by incubating 200 μL of homogenate at 37 °C for 120 min with 50,000 cpm of ^125^I-rT_3_, 0.1 μM unlabeled rT_3_, and 15 mM DTT in 200 μL of 0.01 M PBS (pH 7.0). The activity of ID2 was measured by incubating 200 μL of homogenate at 37 °C for 120 min with 50,000 cpm of ^125^I-T_4_, 1 nM unlabeled T_4_, and 30 mM DTT in 200 μL of 0.01 M PBS (pH 7.0). The activity of ID3 was measured by incubating 200 μL of homogenate at 37 °C for 120 min with 150,000 cpm of ^125^I-T_3_, 1 nM unlabeled T_3_, and 30 mM DTT in 200 μL of 0.01 M PBS (pH 7.0). All reactions were stopped with the successive addition of 200 μL of 5% (*w*/*v*) bovine serum albumin (Sigma-Aldrich, St. Louis, MO, USA) and 400 μL of 10% (*w*/*v*) trichloroacetic acid at 4 °C. Each mixture was centrifuged at 3500× *g* for 30 min, and the radioactivity in the supernatant was counted using a GC-911 γ-counter (Zhong Jia, Tianjin, China). In the blank controls, 0.01 M PBS was used instead of the homogenate and the procedures were performed as described above. The ID activities were calculated using the following formula:

$$\text{Iodothyronine deiodinase activity}=\frac{\left[\mathrm{SCc}(\mathrm{cpm})\times \mathrm{SA}\;(\mathrm{fmol}/\mathrm{cpm})\times 1000\right]}{\left[\text{homogenate volume}(\mu \text{L})\times \text{protein content}(\mathrm{mg}/\mathrm{mL})\times \text{incubation time}(\min)\right]}$$

In which SCc is sample counts minus blank counts and SA is total moles of TH (rT_3_, T_4_, or T_3_) in the incubation solution divided by total counts. Therefore, the units of iodothyronine deiodinase activity are expressed as fmol I^−^ released/mg protein per min.

### 4.7. Statistical Analysis

All data analyses were performed using SPSS 16.0 software (SPSS, Chicago, IL, USA). The differences between the control group and each treatment group were evaluated by one way analysis of variance (ANOVA) followed by the least significant difference (LSD) test where differences were found. A value of *p* < 0.05 was considered statistically significant. All the data were expressed as the mean ± standard deviation (SD).

## 5. Conclusions

In conclusion, our results demonstrate that the stress response induced by MC-LR significantly alters the activities of iodothyronine deiodinases and cause a drop in T_3_ levels in juvenile zebrafish. Moreover, the hyper-stimulation of TH synthesis and secretion, including the hypertrophy and hyperplasia of the thyroid follicle epithelial cells, the elevations in T_4_ levels as well as the up-regulated genes involved in TH synthesis, can be mainly regard as the negative feedback from the hypothalamus and pituitary due to the decreased levels of T_3_. Taken together, these results reveal the potential disruption of MC-LR on the metabolism of thyroid hormones in juvenile zebrafish, led to a hypothyroidism state after exposure to environmentally relevant concentrations of MC-LR.
